# PTK7, a Catalytically Inactive Receptor Tyrosine Kinase, Increases Oncogenic Phenotypes in Xenograft Tumors of Esophageal Squamous Cell Carcinoma KYSE-30 Cells

**DOI:** 10.3390/ijms23042391

**Published:** 2022-02-21

**Authors:** Won-Sik Shin, Mi-Kyung Park, Jae Hoon Kim, Si Won Oh, Ji-Yun Jang, Ho Lee, Seung-Taek Lee

**Affiliations:** 1 Department of Biochemistry, College of Life Science and Biotechnology, Yonsei University, Seoul 03722, Korea; amgod0306@hanmail.net (W.-S.S.); ebahspa@yonsei.ac.kr (J.H.K.); dhtldnjs1993@naver.com (S.W.O.); 2 Department of Cancer Biomedical Science, Graduate School of Cancer Science and Policy, National Cancer Center, Goyang 10408, Korea; mkpark@ncc.re.kr (M.-K.P.); yun9230@ncc.re.kr (J.-Y.J.); 3 College of Pharmacy, Dongguk University, Seoul 04620, Korea

**Keywords:** esophageal squamous cell carcinoma, KYSE-30 cells, oncogene, PTK7, receptor protein tyrosine kinase

## Abstract

Protein tyrosine kinase 7 (PTK7), a catalytically defective receptor protein tyrosine kinase, is upregulated in tumor tissues and cell lines of esophageal squamous cell carcinoma (ESCC). We showed that PTK7 plays an oncogenic role in various ESCC cell lines. However, its role as an oncogene has not been demonstrated in vivo. Here, we examined the influence of PTK7 on the tumorigenic potential of ESCC KYSE-30 cells, which are known to establish xenograft tumors. Overexpression of PTK7 enhanced the proliferation, adhesion, wound healing, and migration of KYSE-30 cells, and these effects were reversed by the knockdown of *PTK7*. *PTK7* overexpression and knockdown, respectively, increased and decreased the tyrosine phosphorylation of cellular proteins and the phosphorylation of ERK, AKT, and FAK, which are important for cell proliferation, survival, adhesion, and migration. Additionally, *PTK7* overexpression and silencing, respectively, increased and decreased the weight, volume, and number of Ki-67-positive proliferating cells in xenograft tumors of KYSE-30 cells. Therefore, we propose that PTK7 plays an important role in the tumorigenesis of ESCC cells in vivo and is a potential therapeutic target for ESCC.

## 1. Introduction

Protein tyrosine kinase 7 (PTK7) (also known as colon carcinoma kinase-4, CCK-4) is a catalytically defective receptor protein tyrosine kinase (RPTK) molecule that contains an extracellular domain with seven immunoglobulin-like loops, a transmembrane domain, and a tyrosine kinase domain lacking catalytic activity [1].

Homozygous *PTK7* knockout mice are perinatally lethal with severe developmental defects, including defective neural tube closure [2]. The *PTK7* knockout mice were phenotypically similar to mice and *Xenopus* with mutations in the planar cell polarity (PCP) genes. *PTK7* is also genetically linked to the PCP gene *Vangl2*. In addition, PTK7 interacts with canonical Wnt signaling pathway proteins, including β-catenin, and activates genes involved in *Xenopus* development, such as the formation of Spemann’s organizer [3]. Moreover, PTK7 functions in non-canonical Wnt signaling by switching off canonical Wnt signaling [4]. PTK7 interacts with Wnt5A, a non-canonical Wnt ligand, and induces morphogenetic cell movements in *Xenopus* [5]. These findings suggest that PTK7 regulates the PCP and canonical and noncanonical Wnt signaling pathways during development.

PTK7 is an important modulator of tumorigenesis in adult tissues and is upregulated in esophageal squamous cell carcinoma (ESCC) [6,7], colorectal cancer [8,9], and other cancers [10,11,12]. PTK7 specifically promotes proliferation, survival, migration, invasion, and wound healing, and decreases apoptosis in many cell types [6,10,13,14,15,16]. However, PTK7 also acts as a tumor suppressor in some cell types, such as epithelial ovarian carcinoma and lung squamous cell carcinoma cells [17,18].

Esophageal cancer (EC) is the seventh most common cancer worldwide and the sixth leading cause of malignancy-related deaths [19]. EC is divided into two major subtypes, namely, esophageal adenocarcinoma (EAC) and ESCC. EAC is the most common subtype in Western countries, and the main risk factors include gastroesophageal reflux disease and obesity [20]. Globally, ESCC is the most common EC, with the highest incidence in East Asia and parts of Africa. Cigarette smoking and alcohol consumption are the main risk factors for ESCC [21,22].

PTK7 is upregulated in ESCC [6,7] and plays a role in ESCC cell proliferation, survival, migration, and invasion [15,16]. Fibroblast growth factor receptor 1 (FGFR1) is frequently upregulated in ESCC tumor tissues and cell lines [23]. PTK7 binds and activates FGFR1 independent of FGF, thereby increasing the tumorigenicity of PTK7- and FGFR1-positive cells [23].

We have shown that PTK7 plays a role in enhancing oncogenic properties in TE-6, 9, 10, and 11 ESCC cells harboring *TP53* mutations [24]. However, TE-10 cells, which showed a distinct PTK7-dependent tumorigenic response [6,15], did not induce xenograft tumors in nude mice (unpublished data). KYSE-30 ESCC cells harboring epidermal growth factor receptor (*EGFR*) overexpression and *TP53* mutations [25,26,27] can generate tumors in xenografts of orthotopic and subcutaneous implantation [28,29]. In this study, we analyzed the role of PTK7 in oncogenic phenotypes and the activation of signaling proteins in KYSE-30 cells using *PTK7* knockdown and overexpression. We then analyzed the role of PTK7 in tumorigenesis in subcutaneous xenograft mice with KYSE-30 cells carrying knocked-down and overexpressed *PTK7*.

## 2. Results

### 2.1. PTK7 Overexpression Increases Proliferation, Adhesion, and Migration in ESCC KYSE-30 Cells

**Figure 1:**
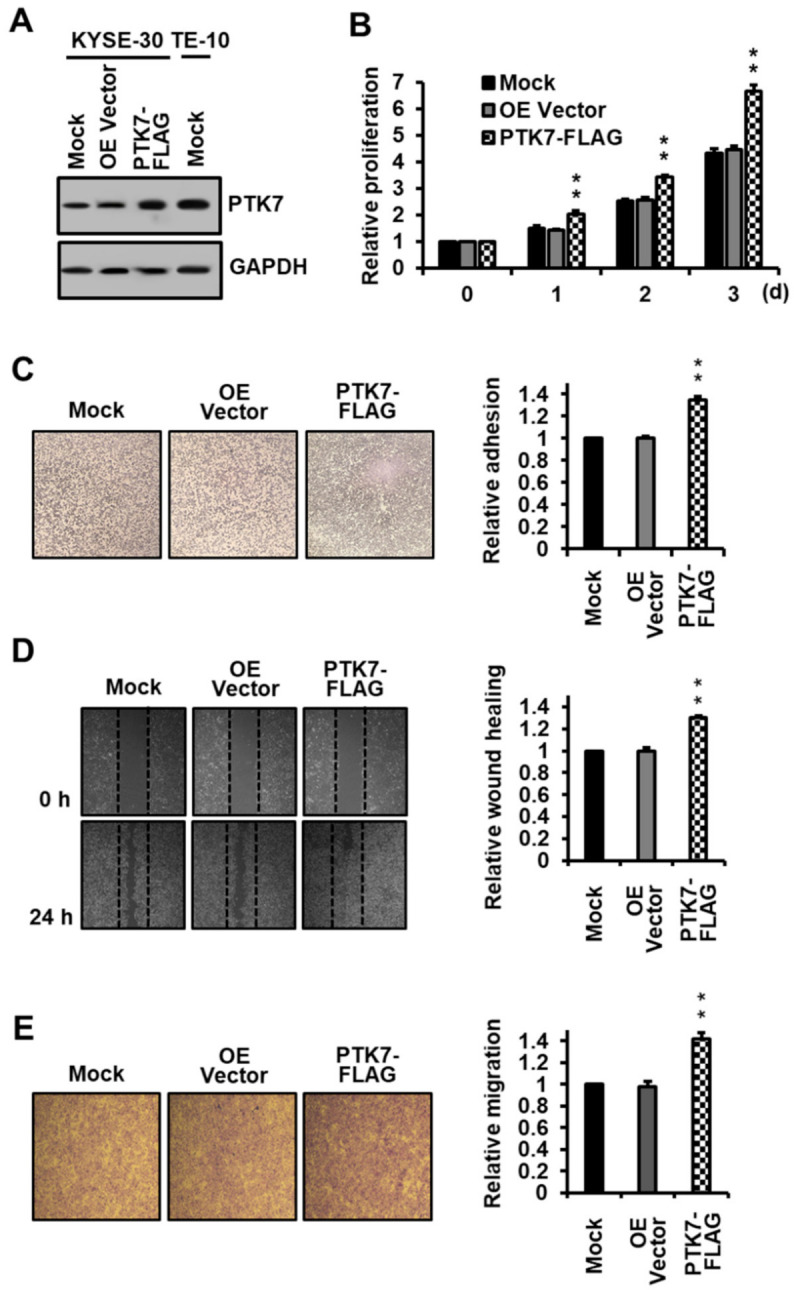
Effect of PTK7 overexpression on the oncogenic properties of ESCC KYSE-30 cells. (**A**) PTK7 expression in ESCC KYSE-30 cells mock-infected or infected with lentiviruses for vector control (OE Vector) and for expression of PTK7-FLAG and in ESCC TE-10 cells mock-infected was analyzed by Western blotting with PTK7 and GAPDH antibodies. (**B**) Proliferation of the mock, vector control, and PTK7-overexpressing KYSE-30 cells was analyzed for 3 days. (**C**) Adhesion of the mock, vector control, and PTK7-overexpressing KYSE-30 cells was analyzed 1 h after plating on dishes coated with type I collagen (1 μg/well). (**D**) Wound healing of the mock, vector control, and PTK7-overexpressing KYSE-30 cells was analyzed 24 h after the wounding in the monolayer of cells. (**E**) Chemotactic migration of the mock, vector control, and PTK7-overexpressing KYSE-30 cells was analyzed 24 h after loading of the cells in the upper transwell chamber. The representative images for (**C**–**E**) are shown. Each bar represents the mean ± SD from three independent experiments. ** *p* < 0.01 vs. 0 day (**B**) or vector control (**C**–**E**).

The effect of PTK7 overexpression on the oncogenic properties of ESCC KYSE-30 cells was first examined. Infecting KYSE-30 cells with a PTK7-FLAG lentivirus increased PTK7 expression compared to that by the overexpression vector control (Figure 1A). PTK7 overexpression increased cell growth to 204.5 ± 11.8%, 342.6 ± 8.3%, and 665.5 ± 22.6% at 1, 2, and 3 days of culture, respectively, compared to that by the overexpression vector control (144.0 ± 3.9%, 256.3 ± 11.1%, and 447.0 ± 19.0% at 1, 2, and 3 days of culture, respectively) (Figure 1B). Moreover, PTK7 overexpression increased adhesion by 131.5 ± 2.5% (Figure 1C), wound healing by 130.1 ± 2.1% (Figure 1D), and migration by 141.6 ± 6.1% (Figure 1E), compared to that by the overexpression vector control. The differences in the values observed in the overexpression vector control and those in the mock control were not statistically significant.

### 2.2. PTK7 Knockdown Reduces Proliferation, Adhesion, and Migration in ESCC KYSE-30 Cells

**Figure 2:**
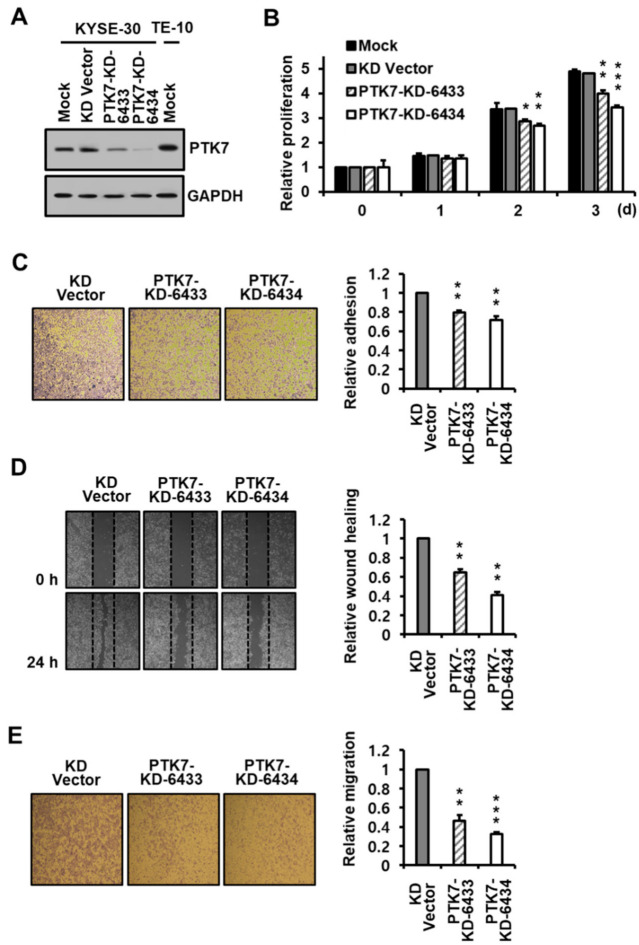
Effect of *PTK7* knockdown on the oncogenic properties of ESCC KYSE-30 cells. (**A**) PTK7 expression in ESCC KYSE-30 cells mock-infected or infected with lentiviruses for vector control (KD Vector) and for *PTK7* knockdown shRNA (PTK7-KD-6433 and PTK7-KD-6434) and in ESCC TE-10 cells mock-infected was analyzed by Western blotting with PTK7 and GAPDH antibodies. Proliferation (**B**), adhesion (**C**), wound healing (**D**), and chemotactic migration (**E**) of the mock, vector control, and *PTK7* knockdown shRNA (PTK7-KD-6433 and PTK7-KD-6434) KYSE-30 cells were analyzed using the same methods as in the overexpressing cells. The representative images for (**C**–**E**) are shown. Each bar represents the mean ± SD from three independent experiments. * *p* < 0.05, ** *p* < 0.01, and *** *p* < 0.001 vs. 0 day (**B**) or vector control (**C**–**E**).

ESCC KYSE-30 cells were infected with lentiviruses encoding *PTK7* shRNAs (PTK7-KD-6433 and -6434) to silence PTK7 expression and examine its effect on the oncogenic properties of these cells. *PTK7* knockdown reduced PTK7 expression in KYSE-30 cells (Figure 2A), and *PTK7* knockdown using PTK7-KD-6433 and -6434 decreased cell proliferation to 398.1 ± 14.4% and 341.9 ± 9.4%, respectively, compared to that by the vector control at 482.1 ± 14.2%, after 3 days of culturing (Figure 2B). *PTK7* knockdown using PTK7-KD-6433 and -6434 also reduced adhesion to 80.9 ± 2.3% and 69.5 ± 1.6% (Figure 2C), wound healing to 65.0 ± 3.1% and 41.2 ± 3.2% (Figure 2D), and migration to 46.4 ± 5.7% and 32.5 ± 2.0% (Figure 2E), respectively, in comparison to that by the knockdown vector control. *PTK7* overexpression and knockdown exhibited an opposing effect on the tumorigenic effect, indicating that PTK7 acts as an oncogene in KYSE-30 cells.

### 2.3. PTK7 Increases the Activation of Oncogenic Signaling Proteins

**Figure 3:**
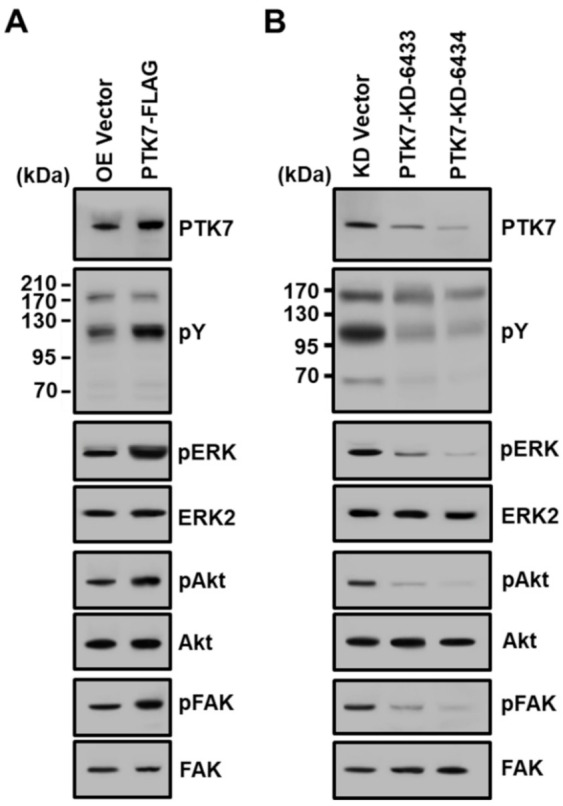
Effect of *PTK7* overexpression and knockdown on activation of signaling molecules in ESCC KYSE-30 cells. Tyrosine phosphorylation of cellular proteins, as well as phosphorylation of ERK, AKT, and FAK, was examined by Western blotting in KYSE-30 cells infected with lentiviruses for vector control (OE Vector) and PTK7 overexpression (PTK7-FLAG) (**A**), as well as the vector control (KD Vector) and *PTK7* knockdown shRNA (PTK7-KD-6433 and -6434) (**B**).

Cell proliferation, adhesion, and migration involve the activation of various signaling pathways, including the MAPK, PI3-kinase/AKT, and FAK pathways [30,31,32]. We investigated the phosphorylation of these signaling proteins in *PTK7*-overexpressing or knockdown KYSE-30 cells. The tyrosine phosphorylation of cellular proteins and the phosphorylation of ERK, AKT, and FAK were increased by PTK7 overexpression and decreased by *PTK7* knockdown (Figure 3). Thus, PTK7 activates the signaling pathways involved in cell proliferation, adhesion, and migration, resulting in increased tumorigenesis of ESCC KYSE-30 cells.

### 2.4. PTK7 Expression Correlates with the Tumorigenic Effect of ESCC KYSE-30 Cells In Vivo

**Figure 4:**
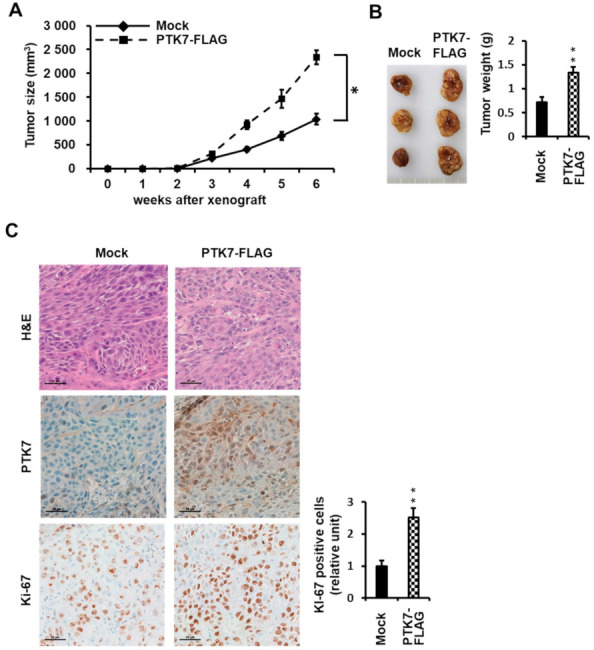
Effect of PTK7 overexpression on tumor growth in xenograft mice. KYSE-30 cells with mock-transfected or overexpressing PTK7-FLAG were injected subcutaneously into the dorsal regions of mice. (**A**) Tumor volumes were monitored once per week for 6 weeks. (**B**) Images of tumors from mice at 6 weeks after injection (left) and the corresponding weights (right) are shown. Each bar represents the mean ± SD of the three mice. * *p* < 0.05, ** *p* < 0.01 vs. mock-transfected cells. (**C**) Representative images of histologic and immunohistochemical (PTK7 and Ki-67) analysis of tumors are shown in the indicated groups of mice. Scale bar represents 50 μm.

**Figure 5:**
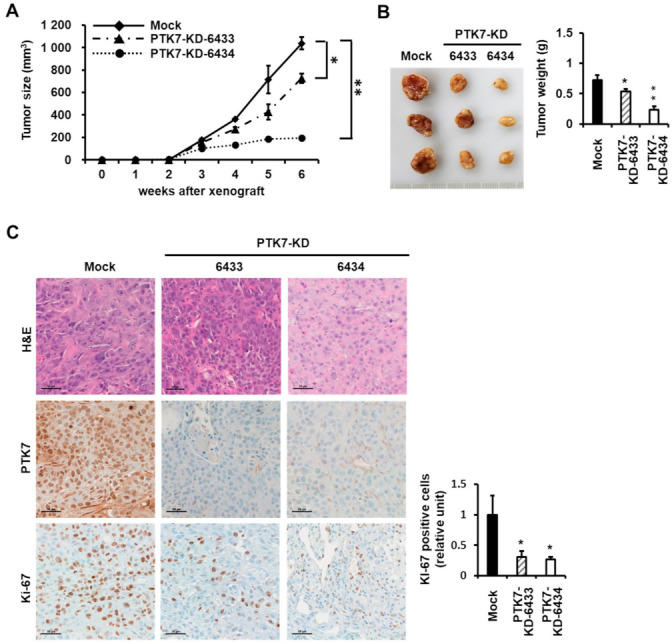
Effect of *PTK7* knockdown on tumor growth in xenograft mice. KYSE-30 cells with mock-transfected or *PTK7* knockdown (PTK7-KD-6433 and -6434) were subcutaneously xenografted into the dorsal regions of mice. (**A**) Tumor volumes were recorded for 6 weeks. (**B**) Images of tumors from mice at 6 weeks after injection (left) and the corresponding weights (right) are shown. Each bar represents the mean ± SD of the three mice. * *p* < 0.05, ** *p* < 0.01 vs. mock-transfected cells. (**C**) Representative images of histologic and immunohistochemical (PTK7 and Ki-67) analysis of tumors are shown in the indicated groups of mice. Scale bar represents 50 μm.

To further elucidate the effect of PTK7 expression on tumorigenesis in vivo, we transplanted mock, PTK7-FLAG, PTK7-KD-6433, or PTK7-KD-6434 KYSE-30 cells into immune-deficient athymic nude (BALB/c nu/nu) mice. Xenografted mice bearing PTK7-overexpressing KYSE-30 cells showed accelerated tumor growth compared to mock-control mice (Figure 4A), while mice harboring *PTK7*-knockdown KYSE-30 cells exhibited a delay in tumor growth compared to control mice (Figure 5A). Mice were sacrificed 6 weeks post injection. The tumor weight increased to 190.5 ± 17.2% in PTK7-overexpressing tumors with PTK7-FLAG (Figure 4B), compared to the tumor weight (0.72 ± 0.11 g) of the mock control tumors. The tumor weight decreased to 73.8 ± 5.2% and 31.7 ± 8.6% in *PTK7*-knockdown tumors with PTK7-KD-6433 and PTK7-KD-6434, respectively (Figure 5B), compared to the tumor weight (0.73 ± 0.08 g) of the mock control tumors.

Hematoxylin and eosin staining and immunohistochemical staining for PTK7 and Ki-67 were performed on the tumor sections to assess changes in cell morphology and proliferation. PTK7 expression was elevated in PTK7-FLAG tumors and decreased in PTK7-KD-6433 and -6434 tumors compared to that in the control tumors (Figure 4C and Figure 5C). Expression of Ki-67, a proliferation marker in solid tumors, was stronger (252.1 ± 29.2%) in PTK7-overexpressing tumors and weaker (30.6 ± 10.1% in PTK7-KD-6433 and 26.8 ± 4.0% in PTK7-KD-6434) in *PTK7*-knockdown tumors, compared to that in control tumors (Figure 4C and Figure 5C). This confirmed that PTK7 promotes tumor progression in ESCC.

## 3. Discussion

Analysis of the biological pathways deregulated in ESCC showed that the RPTK-MAPK-PI3K pathway, cell cycle, and epigenetic regulatory mechanisms are frequently dysregulated by multiple molecular abnormalities [33,34,35]. In ESCC tumor tissues, EGFR is frequently overexpressed or often amplified, with activation of the PI3K/AKT signaling pathway due to mutations in the *PI3KCA* gene and loss of PTEN expression [36,37,38]. *TP53* and *CDKN2A* mutations and *CCND1* amplification are additional changes observed in the genes regulating the cell cycle [39,40]. Mutations are often found in genes involved in epigenetic regulation, such as histone H3 lysine-4 mono-methyltransferases (*KMT2D*/*MLL2* and *KMT2C*/*MLL3*) and histone acetyltransferases (*CREBBP* and *EP300*) [39].

EGFR overexpression and *TP53* mutations are common in precancerous ESCC lesions [36,41]. *TP53* mutations were also correlated with EGFR overexpression. These findings suggest that patients with ESCC may benefit from EGFR-targeted therapy. Nevertheless, the EGFR-neutralizing antibody cetuximab only reacted to ESCC patient-derived xenografts with high EGFR expression and/or amplification [42]. In addition, gefitinib, a low molecular weight EGFR inhibitor, did not improve overall survival in unselected patients with EC, including ESCC, but provided a palliative effect in a subgroup of difficult-to-treat patients with short life expectancy [43]. Another low molecular weight EGFR inhibitor, icotinib, was effective in 17.6% of patients with high EGFR-expressing tumors, but not in patients with moderate EGFR-expressing tumors [44]. EGFR inhibitors are effective for epithelial-like ESCC cells but are ineffective for mesenchymal-like ESCC cells, as EGFR signaling cannot be blocked [45]. EGFR inhibitors alone are, therefore, considered less effective in treating ESCC.

PTK7 is upregulated in various cancer types, including ESCC [6,7]. PTK7 reduces apoptosis and promotes proliferation, survival, migration, invasion, and wound healing in ESCC cells [6,16]. *PTK7* knockdown reduced the phosphorylation of Akt, Erk, and FAK [6]. PTK7 upregulates MMP-9 through activation of AP-1 and NF-κB, thus increasing the invasive properties of ESCC cells [15]. These results suggest that PTK7 has potential as a prognostic marker for ESCC and could be a candidate for targeted therapy.

In our analysis, the PTK7 levels in ESCC TE-10 and TE-11 cells were higher than those in TE-5, TE-9, and TE-14 cells [6]. TE-10 cells exhibited a PTK7-dependent tumorigenic response, including cell proliferation, survival, wound healing, and invasion [6,15]. TE-10 cells harbor a *TP53* mutation [24] and co-amplified *INT-2*/*FGF3* and *HST-1*/*FGF4*. The INT-2/FGF3 and HST-1/FGF4 polypeptides are members of the FGF family, which have mitogenic activity in various cell types [46]. Additionally, *PTK7* knockdown reduced not only ligand-free and FGF-induced FGFR1 phosphorylation, but also the interaction of signaling adaptor proteins with FGFR1 and activation of downstream signaling proteins in TE-10 cells [23]. Collectively, these results suggest that the FGF/FGFR signaling pathway, in cooperation with PTK7, plays an important role in the pathogenesis of EC in an autocrine or paracrine manner.

We also wanted to use a mouse xenograft model of ESCC cells to demonstrate the role of PTK7 in tumorigenesis. However, it was unclear whether TE-10 cells could establish xenograft tumors in nude mice. Nishihara et al. reported that xenograft tumors could not be generated [46], while Yang et al. recently reported that xenograft tumors were produced using TE-10 cells [47]. However, we were unable to generate tumors after performing xenografts in nude mice with TE-10 cells (unpublished data).

KYSE-30 cells were established in nude mice tumors generated through transplantation of well-differentiated invasive ESCC tissue from a 64-year-old Japanese man [26]. KYSE-30 cells have a highly rearranged hypertriploid karyotype with 8% polyploidy and harbor *TP53* mutations and amplification of *EGFR*, *MYC*, and *CCND1*. Additionally, KYSE-30 cells produce xenograft tumors in nude mice [48,49].

In this study, we showed that KYSE-30 cells express PTK7 at lower levels relative to TE-10 cells. Consistent with our previous results using other ESCC cells [6,15,50], PTK7 overexpression increased proliferation, adhesion, wound healing, and migration as well as tyrosine phosphorylation of cellular proteins and phosphorylation of ERK, AKT, and FAK in KYSE-30 cells. When *PTK7* was silenced in KYSE-30 cells, these effects were reversed. ERK inhibition by PD98059 (MEK inhibitor) and Akt inhibition by LY294002 (PI3K inhibitor) in ESCC TE-10 cells, in which PTK7 expression increases invasion via MMP-9 secretion, decreased MMP-9 secretion [15]. Similarly, ERK inhibition by PD98059 and Akt inhibition by LY294002 significantly suppress migration and invasion in ESCC TE-8 and TE-9 cells [51]. Furthermore, knockdown or inhibition (by defactinib) of FAK decreases proliferation, migration, and invasion in various ESCC cells, including KYSE-30 cells [52,53]. Therefore, PTK7-dependent activation of ERK, Akt and FAK appears to be directly related to the oncogenic phenotypes.

Various studies have shown that PTK7 and FGFR1 are significantly upregulated in ESCC tumor tissues and cell lines [6,23,54]. We also demonstrated that PTK7 plays an important role in FGFR1 activation in various ESCC cells [23]. Therefore, regulation of FGFR1 activity, based on PTK7 expression, could possibly modulate oncogenic phenotypes and signaling pathways. However, considering the high EGFR levels in KYSE-30 cells, it was interesting how *PTK7* knockdown could reduce oncogenic processes. It was recently reported that PTK7 is involved in the activation of EGFR and Akt signaling in triple-negative breast cancer cells [55]. These results suggest that counteracting PTK7 can efficiently reduce the tumorigenesis of esophageal squamous cells in ESCC cells by blocking FGFR and EGFR signaling.

Importantly, we could demonstrate that xenograft tumors of KYSE-30 cells with *PTK7* overexpression and silencing respectively increased and decreased the weight, volume, and number of Ki-67-positive proliferating cells. This proved that PTK7 expression is positively correlated with the tumorigenic process of ESCC in vivo. In addition, this xenograft tumor model of KYSE-30 cells can be used to analyze anti-cancer agents targeting PTK7 and the role of PTK7 in vivo.

## 4. Materials and Methods

### 4.1. Cell Culture

Human ESCC KYSE-30 cells and human embryonic kidney 293 (HEK293) cells expressing SV40 T antigen (HEK293T) were provided by Dr. Sang Kil Lee (Yonsei University College of Medicine, Seoul, Korea) and Dr. Jong Bae Park (National Cancer Center, Goyang, Gyeonggi, Korea), respectively. KYSE-30 cells were grown in Dulbecco’s modified Eagle medium/nutrient mixture F-12 medium (DMEM/F12; Gibco of Thermo Fisher Scientific, Grand Island, NY, USA), supplemented with 2.5 mM sodium glutamine and 2% fetal bovine serum (FBS). HEK293T cells were grown in DMEM supplemented with 10% bovine serum (BS). All media were supplemented with 100 U/ml penicillin and 100 μg/mL streptomycin, and all cells were maintained at 37 °C in 5% CO_2_.

### 4.2. Generation of PTK7 Overexpression and Knockdown Lentiviruses and Infection of KYSE-30 Cells

To construct the pHRST-PTK7-FLAG-IRES-eGFP lentiviral transfer vector for PTK7 expression, a DNA fragment including the PTK7-FLAG-coding sequence was amplified by polymerase chain reaction (PCR), using the pcDNA3-hPTK7-FLAG vector [56] as a template, and PrimeSTAR GXL DNA polymerase. The upstream primer (5′-GATCTCGACGCGGCCGC*TACGACTCACTATAGGGAGACCCAAGCT*-3′) contained 17 nucleotides of pHRST-IRES-eGFP (nt. 2839-2855), including a NotI site (underlined) and 28 nucleotides of pcDNA3 (nt. 866-893; italicized). The downstream primer (5′-GGGCGGAATTGGATCC*GAAGGCACAGTCGAGGCTGATCAG*-3′) contained 16 nucleotides of pHRST-IRES-eGFP (nt. 2871-2856), including a BamHI site (underlined) and 24 nucleotides of pcDNA3 (nt. 1028-1051; italicized). The PCR product was ligated into the NotI and BamHI sites of the pHRST-IRES-eGFP vector using the In-fusion HD cloning kit (TaKaRa, Kusatsu, Japan). The resulting construct, pHRST-PTK7-FLAG-IRES-eGFP, was bi-directionally sequenced to avoid PCR errors. The pLKO.1-shRNA-PTK7-6433 and -6434 constructs for human *PTK7* knockdown and the pLKO.1-control were purchased from Sigma-Aldrich (St. Louis, MO, USA). Lentiviruses were produced in HEK293T cells, as previously described [6], with the exception of using 13 μg of pHRST-PTK7-FLAG-IRES-eGFP for *PTK7* overexpression. KYSE-30 cells infected with *PTK7* knockdown lentiviruses were incubated with 1 μg/ml puromycin for 14 days, and puromycin-resistant *PTK7*-knockdown colonies were pooled in a mixed culture. Cells infected with *PTK7*-overexpression lentiviruses were used without further selection.

### 4.3. Antibodies

The following antibodies were used: anti-phospho-ERK (sc-7383) and anti-FAK (sc-557), obtained from Santa Cruz Biotechnology (Santa Cruz, CA, USA); anti-phospho-Akt (Ser473) (4060s) and anti-Akt (9272s), obtained from Cell Signaling Technology (Danvers, MA, USA); anti-phospho-tyrosine (clone 4G10) (05-321) and anti-phospho-FAK (Tyr397) (abt135), obtained from Merck Millipore (Burlington, MA, USA); anti-ERK2 (bms-52068R), obtained from Bioss (Boston, MA, USA); anti-FLAG-M2 antibody (F1804), obtained from Sigma-Aldrich; anti-Ki-67 (ab15580), obtained from Abcam (Cambridge, UK); anti-GAPDH (abc2003), obtained from AbClone (Seoul, Korea); and horseradish peroxidase-conjugated goat anti-mouse IgG (K0211589) and rabbit IgG (K0211708), obtained from KOMA Biotech (Seoul, Korea). The generation of anti-PTK7 anti-serum was described previously [57].

### 4.4. Western Blot Analysis

For Western blot analysis, cells were lysed with RIPA buffer (50 mM Tris-HCl, pH 7.4, 150 mM NaCl, 1% NP-40, 0.5% sodium deoxycholate, and 0.1% SDS) for 10 min at 4 °C. Lysates were subjected to SDS-PAGE and transferred onto a polyvinylidene difluoride membrane. Blots were incubated with the indicated antibodies, and the immunoreactive bands were visualized using West-Q PICO Dura ECL solution (GenDepot, Barker, TX, USA), Immobilon Western Chemiluminescent HRP Substrate (Merck Millipore), and an LAS-3000 imaging system (Fujifilm, Tokyo, Japan).

### 4.5. Cell Proliferation Assay

Cell proliferation assays were performed as previously described [58]. Briefly, cells (0.1 ml, 1.5 × 10^4^ cells/ml) were plated in a 96-well flat-bottomed cell culture plate (SPL, Pocheon, Korea) in DMEM/F12 medium supplemented with 2% FBS and 2.5 mM sodium glutamine and incubated for 1–3 days. To measure the number of live cells, cells were washed with phosphate-buffered saline (PBS; 8.06 mM Na_2_HPO_4_, 1.47 mM KH_2_PO_4_, 137 mM NaCl, 2.6 mM KCl; pH 7.4) containing 1 mM CaCl_2_ and 0.5 mM MgCl_2_ and incubated with 0.1 ml DMEM/F12 medium containing 0.5 mg/ml 3-(4,5-dimethylthiazol-2-yl)-2,5-diphenyltetrazolium bromide for 4 h. After removal of the medium, cells were washed with PBS and solubilized with 0.1 ml DMSO. Absorbance was measured at 565 nm using a SpectraMax M3 microplate reader (Molecular Devices, San Jose, CA, USA).

### 4.6. Cell Adhesion Assay

The cell adhesion assay was performed as previously described [59]. Briefly, cells were incubated in serum-free DMEM/F12 medium for 24 h. Detached cell suspensions (3 × 10^4^ cells/0.1 mL for overexpression analysis or 5 10^4^ cells/0.1 mL for knockdown analysis) were loaded onto 96-well plates, which were precoated with rat-tail type I collagen (1 μg/well) overnight and were incubated in DMEM/F12 medium with 1% FBS for 1 h. Cells were fixed with 3.7% paraformaldehyde in PBS and stained with 0.005% crystal violet. The stained cells were lysed with 1% SDS, and the absorbance was measured at 600 nm.

### 4.7. Wound Healing and Chemotactic Migration Assay

A wound was introduced by scraping the monolayer with a micropipette tip. Cells were incubated for 24 h in DMEM/F12 medium containing 2% FBS and evaluated by light microscopy [59]. The chemotactic migration assay was performed as previously described [50]. Briefly, detached cell suspensions (7 × 10^4^ cells/0.1 mL serum-free DMEM/F12 medium) were loaded into the upper compartment of transwell chambers (Corning, Tewksbury, MA, USA). The bottom surface of each transwell was coated with 10 μL of 0.1% gelatin. The lower compartment of each well was filled with 0.65 mL DMEM/F12 medium, with 10% FBS as a chemoattractant. The chamber was then incubated at 37 °C for 24 h. After incubation, the remaining cells in the upper compartment were removed using a cotton swab. Cells that migrated to the bottom surface of the filter were fixed with 3.7% paraformaldehyde in PBS and stained with 0.005% crystal violet. The stained cells were solubilized with 1% SDS, and the absorbance was measured at 600 nm.

### 4.8. Xenograft Mouse Model

Four to five-week-old male immune-deficient athymic nude (BALB/c nu/nu) mice were purchased from Orient Bio Inc. (Gyeonggi, Korea). To investigate the effect of PTK7 on tumorigenesis in a xenograft mouse model, 1 × 10^6^ KYSE-30 cells expressing either PTK7-FLAG or *PTK7* shRNA (PTK7-KD-6433 and 6434) were resuspended in growth-factor-reduced Matrigel (Corning) and subcutaneously injected into the backs of mice. Tumor growth in each group was evaluated by measuring the tumor size twice per week using calipers (length × width × depth/2). Mice were sacrificed 6 weeks after cell injection. The xenograft tumors were recovered to measure tumor weight, fixed in formalin, and were paraffin embedded for histological and immunohistochemical analyses [60].

### 4.9. Ethics Statement

All animal experiments were approved and performed in accordance with the Institutional Animal Care and Use Committee (IACUC) review board of the National Cancer Center, which is an Association for Assessment and Accreditation of Laboratory Animal Care International (AAALAC International) accredited facility that abides by the Institute of Laboratory Animal Resources guide (NCC-20-561).

### 4.10. Statistical Analysis

Statistical analyses were performed using Microsoft Excel (Microsoft Corp., Redmond, WA, USA). Statistical significance was analyzed using the Student’s *t* test. For all tests, *p* values less than 0.05 were considered statistically significant.

## 5. Conclusions

We previously showed that PTK7 expression correlates with the promotion of oncogenic properties in several ESCC cells. In this study, we analyzed the effect of PTK7 on tumorigenicity at the cellular level and in vivo using KYSE-30 cells, which are known to produce xenograft tumors in nude mice. KYSE-30 cells expressed PTK7 at a relatively lower level than TE-10 cells. Overexpression of PTK7 increased proliferation, adhesion, wound healing, and migration, as well as tyrosine phosphorylation of cellular proteins and phosphorylation of ERK, AKT, and FAK in KYSE-30 cells. *PTK7* silencing in KYSE-30 cells was reversed by PTK7 overexpression. *PTK7* overexpression and silencing increased and decreased, respectively, the weight, volume, and number of Ki-67-positive proliferating cells in xenograft tumors of KYSE-30 cells. The results showed that the expression of PTK7 in vivo correlates positively with the oncogenic process of ESCC. Furthermore, a xenograft tumor model using KYSE-30 cells can be used to analyze PTK7-targeted anti-cancer drugs and the role of PTK7 in vivo.

## Data Availability

Not applicable.

## Abbreviations

DMEM	Dulbecco’s modified Eagle medium
EAC	esophageal adenocarcinoma
EC	esophageal cancer
EGFR	epidermal growth factor receptor
ESCC	esophageal squamous cell carcinoma
FBS	fetal bovine serum
FGFR	fibroblast growth factor receptor
HEK293	human embryonic kidney 293
PBS	phosphate-buffered saline
PCP	planar cell polarity
PCR	polymerase chain reaction
PTK7	protein tyrosine kinase 7
RPTK	receptor protein tyrosine kinase
